# Impact of the COVID-19 pandemic on primary brain tumor incidence and management: Decisions that went right

**DOI:** 10.1093/noajnl/vdaf181

**Published:** 2025-08-16

**Authors:** Melanie Alfonzo Horowitz, Megan Parker, Ryan Gensler, Elizabeth Wang, Alyssa Arbuiso, Karisa C Schreck, Kristin J Redmond, Debraj Mukherjee, Jordina Rincon-Torroella

**Affiliations:** Department of Neurosurgery, Johns Hopkins University School of Medicine, Baltimore, Maryland; Department of Neurosurgery, Johns Hopkins University School of Medicine, Baltimore, Maryland; Department of Neurosurgery, Georgetown University School of Medicine, Washington DC; Department of Neurosurgery, Johns Hopkins University School of Medicine, Baltimore, Maryland; Department of Neurosurgery, Johns Hopkins University School of Medicine, Baltimore, Maryland; Department of Neurology, Johns Hopkins University School of Medicine, Baltimore, Maryland, USA; Department of Radiation Oncology and Molecular Radiation Sciences, Johns Hopkins University School of Medicine, Baltimore, Maryland, USA; Department of Neurosurgery, Johns Hopkins University School of Medicine, Baltimore, Maryland; Department of Neurosurgery, Johns Hopkins University School of Medicine, Baltimore, Maryland

**Keywords:** brain tumor, COVID-19, incidence, pandemic, treatment patterns

## Abstract

**Background:**

The COVID-19 pandemic drastically altered cancer care. Prior reports demonstrated reduced screenings, diagnoses, and disrupted treatment regimens due to multifactorial reasons. We aim to analyze whether the same effects occurred within neuro-oncology.

**Methods:**

This analysis included 70 131 patients with primary brain tumors from the SEER database from 2016 to 2021 identified via ICD10 code. The pre-COVID era was 2016-2019, peak-COVID was 2020, and post-COVID was 2021. Multivariate analysis was performed using logistic regression for binary variables and linear regression for continuous. Covariates controlled for were age at diagnosis, sex, and race. NCI SEER*Stat version 8.4.0 was used to calculate incidence rates age-adjusted to the 2000 US standard population and reported per 100 000 persons.

**Results:**

Although there was a decrease in the age-adjusted incidence of primary brain tumors between 2016 and 2021, the number of malignant brain tumors remained stable, and this change was likely driven by a reduction in benign tumor incidence. Regarding treatment, in 2020 and 2021 all malignant brain tumors (2020 OR[95%CI]: 1.11[1.02-1.22], 2021: 1.10[1.01-1.020]) and glioblastoma patients (2020 OR[95%CI]: 1.12[1.01-1.26], 2021: 1.13[1.01-1.27]) underwent increased surgical resections, compared to pre-COVID years. Time from diagnosis to treatment decreased for glioblastoma patients in 2020, compared to pre-COVID (Estimate [95%CI]: −1.25 [−1.71 to −0.78]). No treatment changes were noted for benign brain tumors.

**Conclusion:**

Malignant tumors, like glioblastoma, maintained a stable incidence due to their aggressive symptoms, though treatment patterns shifted. These findings reveal that the management of malignant brain tumors during the COVID-19 pandemic was effectively prioritized while maintaining quality of care.

**Key Points:**

- Malignant brain tumor incidence remained stable during the COVID-19 pandemic.
- Treatment patterns shifted, with quicker time to surgery and increased chemotherapy use.
- Healthcare systems prioritized aggressive brain tumors despite pandemic strain.

**Importance of the Study**

While prior studies have shown widespread delays and declines in cancer care during the COVID-19 pandemic, few have focused on how brain tumors were affected. Using national SEER data from 2016 to 2021, this study found that, unlike many other tumor types, the incidence of malignant brain tumors remained stable during the pandemic, suggesting that patients with aggressive neurological symptoms continued to receive diagnostic evaluation. Furthermore, patients experienced faster time to surgery. These findings suggest that despite major system disruptions, high-grade CNS tumors remained a clinical priority. Compared to literature focused broadly on cancer care delays, our data highlight the resilience and adaptability of neuro-oncology practice. These insights may inform future crisis planning and emphasize the importance of preserving timely access to care for patients with rapidly progressive neurologic malignancies.

The COVID-19 pandemic led to national shifts within the structure of United States (US) healthcare systems and its delivery. A conglomeration of COVID-19 contagion, resource constraints, and physician burnout resulted in lower rates of utilization by patients.^1–4^ Due to COVID-19 concerns, many patients sought telehealth visit opportunities and postponed seeking in-person appointments.^5,6^ Additionally, national lockdowns resulted in changes in employment status, resulting in decreased health insurance coverage and ability to afford healthcare.^7^ These healthcare pattern alterations led to increases in overall population morbidity and mortality as individuals avoided seeking medical care, or could not receive protective measures during the lockdown, such as vaccines.^8^

These healthcare system disruptions severely affected cancer care. Due to limitations and avoidance of in-person care, cancer screening rates plummeted 90%.^9^ This included, but is not limited to, mammograms, fecal occult blood, and pap smears. As a result, cancer diagnoses experienced a 65% drop.^10^ This decrease in cancer diagnoses led to significant decreases in the number of treatments provided to tumor patients.^11^ Although some reports state that the time to treatment of newly diagnosed tumors remained stable, treatment regimens were altered.^11,12^ Examples of this include a shift in initial therapy toward the use of preoperative hormonal therapy in breast cancer patients,^12^ oncologists opting for regimens with lower efficacy but lower risk of hospitalization,^13^ and a reduction in the usage of antineoplastic systemic therapies.^14^ In a study of lung cancer patients seen at McGill University Health Centre in 2020, over half experienced changes in their lung cancer treatment plan.^15^ Moreover, in a National Health Interview Survey of 574 adults, one-third experienced disruptions to cancer care due to the COVID‐19 pandemic, including appointment changes, delays, or cancellations.^16^ With lack of identification and prompt management, and cancer patients being more vulnerable to COVID-19, cancer deaths rose by 3.2% during 2020.^17^

Limited investigation has been conducted to determine the effects that COVID-19 had on central nervous system (CNS) tumors and management and whether the effects were similar to those of other oncology fields.^18–20^ Although the lifetime risk of developing a CNS neoplasm is low, in addition to significantly altering quality of life, the overall 5-year survival rate for primary brain tumors is 33.4%.^21^ Early identification followed by prompt treatment and longitudinal monitoring is critical to improve survival time and quality of life in these patients. However, these tumors may follow similar patterns as other cancers did during the COVID-19 pandemic. Therefore, we aim to investigate the effects that the COVID-19 pandemic had on primary brain tumor incidence and treatment patterns using the Surveillance, Epidemiology, and End Results (SEER) database.

## Methods

### Data Collection

We retrieved data from the SEER Research Plus, 17 registries, November 2023 submission, which includes data from 17 population-based registries, representing approximately 26.5% of the US population.^22^ Clinical and demographic data at the time of diagnosis and survival data were collected for all patients. Deidentified data were made publicly available via the NCI SEER program. This study was completed according to the Strengthening the Reporting of Observational Studies in Epidemiology (STROBE) reporting guidelines and approved by The Johns Hopkins Hospital institutional review board.

**Figure 1.**
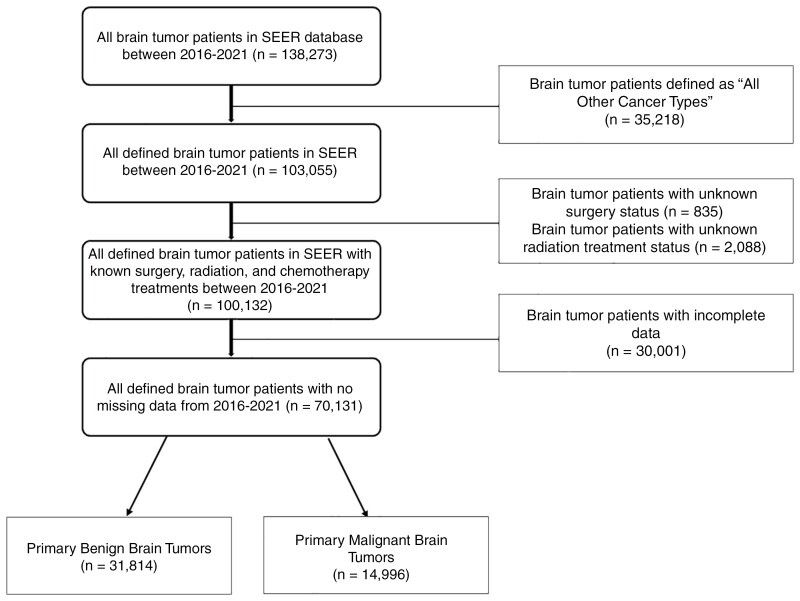
Process for final primary brain tumor cohort selection.

To build our cohorts of patients for our analysis of treatment patterns, we identified 138 237 patients diagnosed with primary brain tumor between January 1, 2016 and December 31, 2021 based on SEER codes (**Supplementary Table 1**). Patients with “all other cancer types” noted for the type of primary brain tumor were excluded. Patients with “borderline malignant,” or “in situ tumors” were excluded. We also excluded patients who had unknown surgery status and unknown radiation status. Data on patients’ follow-up and survival time were required for our survival analyses. NCI excludes patients with 0 days of survival from the SEER database. Patients diagnosed by autopsy or death certificate alone, as well as patients with unknown follow-up were excluded. Finally, we excluded 30 001 patients with missing data for tumor size and time from diagnosis to treatment. Our final cohort consisted of 70 131 patients diagnosed with a primary brain tumor between 2016 and 2021. **Figure 1** depicts our cohort selection process.

Demographics included age at diagnosis, race, sex, and marital status. Patients were also stratified by cancer type, according to the SEER Brain and CNS Record variable, which characterizes primary tumor sites according to the 2016 World Health Organization Classification of Tumors of the Central Nervous System.

### Statistical Analyses

We used the NCI SEER*Stat version 8.4.0 to calculate incidence rates age-adjusted to the 2000 US standard population and reported per 100 000 persons, based on the availability of US Census data in the SEER*Stat software and previous SEER-based studies. The Joinpoint Trend Analysis Software, version 5.2.0 was used to calculate average annual percent changes (AAPC) in incidence rates. The Joinpoint Trend Analysis Software was also used to generate plots of the age-adjusted incidence by year, which include a “joinpoint” or inflection point where the AAPC changes from positive to negative or vice versa. Joinpoints are determined by the Joinpoint software and cannot be adjusted.

All statistical analyses were performed using RStudio statistical software, version 3.3.2 (The R Foundation, Vienna, Austria). Numerical, categorical, and time-to-event variables were compared with Wilcoxon Rank Sum, Chi-squared tests, and log-rank tests, respectively. One-way ANOVA was used to compare means between 3 groups. We compared demographic and treatment patterns for patients diagnosed with primary brain tumors between 3 time categories, 2016-2019 (pre-COVID), 2020 (peak COVID), and 2021 (COVID recovery), which represent 3 historically significant timepoints—the years preceding the first laboratory-confirmed case of COVID-19 in the United States, the year after the first laboratory-confirmed case of COVID-19 in the United States and prior to the release of the COVID-19 vaccine, and the year following the release of the COVID-19 vaccine, respectively. Data for 2022 and after were not yet available at the time of completion of this manuscript. Multivariate logistic regression analyses were performed to evaluate the odds of receiving surgery, radiation, or chemotherapy between each of these timepoints for values significant on univariate analysis. Multivariate linear regression analyses were utilized to evaluate the impact of each these timepoints on the time between diagnosis and treatment of primary brain tumors and tumor size at diagnosis. Covariates utilized in multivariate analyses included age at diagnosis, sex, and race. A value of *P* < 0.05 was considered statistically significant.

## Results

### Cohort Demographics

In total, our final cohort consisted of 70 131 patients diagnosed with new onset primary brain tumors between 2016 and 2021 (**Table 1**). Of these, 47 459 were benign tumors and 22 672 were malignant. Regarding the benign tumors, during the pre-COVID years, 2016-2019, there were a total of 31 814 patients diagnosed (7 593 cases on average, per year). When the pandemic was at its peak in 2020, there were 7 572 patients diagnosed in 2020, which increased to 8 073 patients in 2021 during the COVID-19 recovery phase. For the malignant tumors, during the pre-COVID years, 2016-2019, there were a total of 14 996 patients diagnosed (3 749 cases on average, per year). When the pandemic was at its peak in 2020, there were 3 875 patients diagnosed in 2020, which decreased to 3 801 patients in 2021 during the COVID-19 recovery phase. For the whole cohort of primary brain tumor patients, the median age of patients at diagnosis was around 63 years during 2016-2019 years, and rose to 64 years for 2021. Most patients were male, white, and not Hispanic and/or Latino across all years. Around half of all patients were married at the time of diagnosis across all years.

**Table 1.** Demographic information of all primary brain tumor patients and number of malignant tumors diagnosed, by year between 2016 and 2021.

	Whole cohort (*n* = 22 672)	2016-2019 (*n* = 14 996)	2020 (*n* = 3 875)	2021 (*n* = 3 801)
**Age in years (**median [IQR])	62 [50-72]	62 [50-72]	63 [50.5-72]	63 [50-73]
**Sex**				
Male	13 003 (57.5%)	8 684 (57.9%)	2 260 (58.3%)	2 095 (55.1%)
Female	9 669 (42.7%)	6 348 (42.3%)	1 615 (41.7%)	1 706 (44.9%)
**Race**				
White	19 488 (86.1%)	12 974 (86.5%)	3 280 (84.6%)	3 234 (85.1%)
African American	1 407 (6.2%)	915 (6.1%)	252 (6.5%)	240 (6.3%)
Asian	1 490 (6.6%)	930 (6.2%)	293 (7.6%)	267 (7.0%)
American Indian or Alaska	133 (0.6%)	84 (0.6%)	23 (0.6%)	26 (0.7%)
Unknown	154 (0.7%)	93 (0.6%)	27 (0.7%)	34 (0.9%)
**Ethnicity**				
Not Hispanic/Latino	19 440 (85.9%)	12 988 (86.6%)	3 318 (85.6%)	3 223 (84.8%)
Hispanic/Latino	3 232 (14.3%)	2 097 (14.0%)	557 (14.4%)	578 (15.2%)
**Marital Status**				
Married	13 527 (59.8%)	8 970 (59.8%)	2 320 (59.9%)	2 237 (58.9%)
Unmarried	8 475 (37.5%)	5 593 (37.3%)	1 460 (37.7%)	1 467 (38.6%)
Unknown	625 (2.8%)	433 (2.9%)	95 (2.5%)	97 (2.6%)
**Tumor size in cm** (Median [IQR])	44 [30-57]	44 [30-57]	44 [30-56]	44 [30-57]
**Tumor pathology**	**2016-2019**	**2016-2019** Cases/year	**2020**	**2021**
All Benign	31 814	7 953	7 572	8 073
Meningioma	30 465	7 616	7 230	7 729
Other	1 349	337	342	344
All Malignant	14 996	3 749	3 875	3 801
Glioblastoma	10 239	2 560	2 771	2 639
Oligodendroglioma	881	220	169	188
Diffuse astrocytoma/anaplastic astrocytoma	1 781	445	389	421
High Grade Glioma, other	1 112	278	289	287
Other	983	246	257	266

### Tumor Incidence

**Figure 2.**
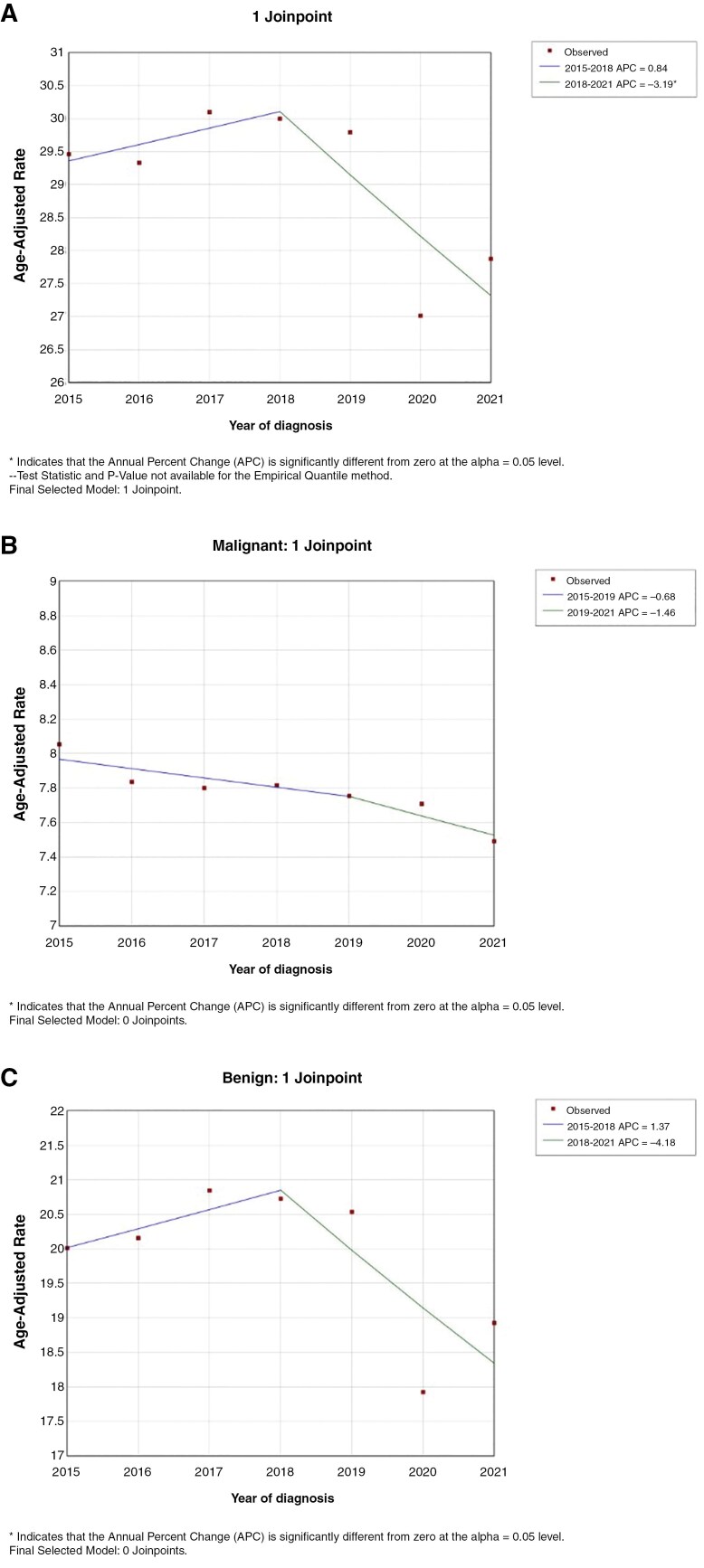
Changes in the age-adjusted incidence rates between 2016 and 2021 for (A) all primary brain tumors, (B) all primary malignant brain tumors, and (C) all primary benign brain tumors. An asterisk by the APC indicates significance of the trend.

Compared to 2016-2019, there was a decrease in age-adjusted incidence of all new primary brain tumors in 2020 (**Figure 2A**). However, when focusing on only primary malignant brain tumors, the age-adjusted incidence of new primary malignant tumors remained stable between 2016 and 2021 (**Figure 2B**). There is a larger drop in the number of primary benign brain tumors diagnosed between 2016 and 2021, although still insignificant (**Figure 2C**). The number of new primary brain tumor diagnoses per time category, classified into subtypes, are listed in **Table 1**.

**Figure 3.**
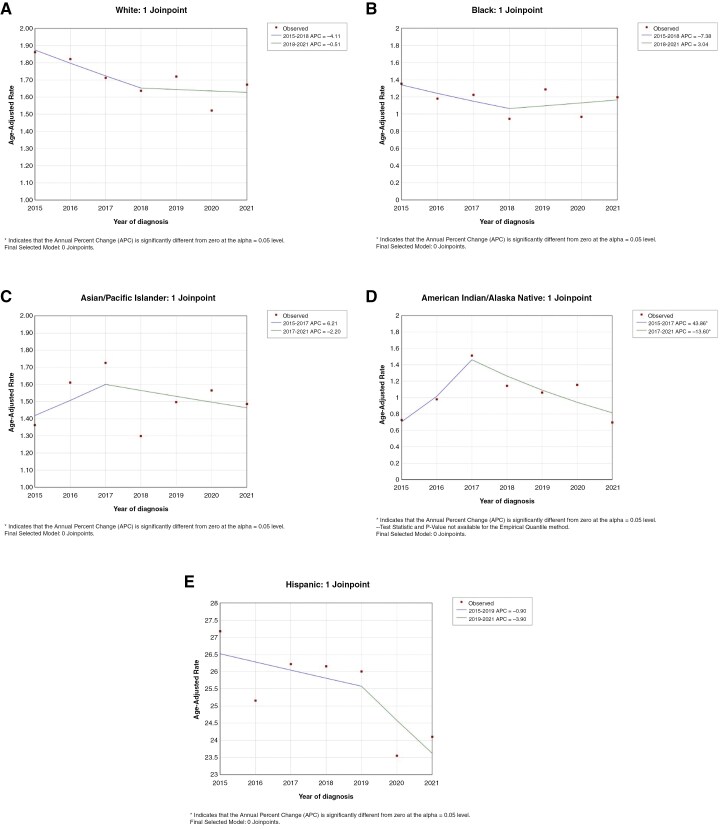
Changes in the age-adjusted incidence rates between 2016 and 2021 for all primary brain tumors in (A) White, (B) Black/African American, (C) Asian/Pacific Islander, (D) American Indian/Native Alaskan, and (E) Hispanic/Latino patients.

When separating age-adjusted incidences by race, there was a significant decrease in number of new diagnoses of all brain tumors combined for American Indian/Alaskan Native patients (**Figure 3D**). When separating by ethnicity, Hispanic/Latino patients had a decrease in incidence, though not significant (**Figure 3E**).

### Trends in Treatment Options

There were no significant changes in treatment patterns for benign primary brain tumors. Regarding surgical resections, there was a decrease in procedures for malignant astrocytoma patients in 2020 compared to 2016-2019, which rose in 2021 (2016-2019: 78.4%, 2020: 76.6%, 2021: 84.4%, *P* = 0.0058) on univariate analysis (**Table 2**). Malignant oligodendroglioma patients experienced an opposing effect, where the number of surgical resections performed in 2020 was higher than pre-COVID and then dropped lower than pre-COVID levels in 2021 (2016-2019: 90.6%, 2020: 92.9%, 2021: 84.6%, *P* = 0.0179). On multivariate analysis, there was an increase in the number of all malignant brain tumors (2020 OR[95%CI]: 1.11 [1.02-1.22], 2021 OR[95%CI]: 1.10 [1.01-1.20]) and glioblastoma patients (2020 OR[95%CI]: 1.12 [1.01-1.26], 2021 OR[95%CI]: 1.13 [1.01-1.27]) who underwent a surgical resection in 2020 and 2021, compared to pre-COVID years (**Table 2**). In 2021, malignant oligodendrogliomas saw a decrease in the number of surgeries (0.60 [0.36-0.91]).

**Table 2.** Number of patients that received surgery, radiation, or chemotherapy treatment separated by primary brain tumor type and year. Multivariate analysis of changes in primary brain tumor treatment patterns in 2020 and 2021, compared to pre-COVID years is shown for treatments with a *P*-value < 0.10 in the univariate analysis. Covariates include age at diagnosis, sex, and race. Reference year was 2016-2019.

	2016-2019 (*n* = 14 996)	2020 (*n* = 3 875)	2021 (*n* = 3 801)	*P*-value	2020 (*n* = 3 875)	*P*-value	2021 (*n* = 3 801)	*P*-value
	*N* (%)				OR [95%CI]:		OR [95%CI]:	
**Surgery**								
All tumors	19 355 (41.4)	4 841 (42.3)	4 879 (41.1)	0.1246				
All benign	7 776 (24.4)	1 786 (23.6)	1 901 (23.6)	0.1113				
Meningioma	7 294 (23.9)	1 654 (22.9)	1 784 (23.1)	0.0741				
All malignant	11 579 (77.2)	3 055 (78.8)	2 978 (78.4)	0.0534	1.11 [1.02-1.22]	0.013	1.10 [1.01-1.20]	0.034
Glioblastoma	8 214 (80.2)	2 271 (81.0)	2 146 (81.3)	0.084	1.12 [1.01-.26]	0.040	1.13 [1.01-1.27]	0.030
Diffuse astrocytoma/anaplastic astrocytoma	1 396 (78.4)	298 (76.6)	357 (84.8)	0.0058	3.8 [1.0-6.60]	0.172	0.07 [0.01, 1.11]	0.336
Oligodendroglioma	798 (90.6%)	157 (92.9%)	159 (84.6%)	.0179	1.34 [0.74-2.64]	0.369	0.60 [0.36-0.91]	0.016
**Radiation**								
All tumors	12 209 (26.1)	3 045 (26.6)	3,031 (25.5)	0.0983				
All benign	1 652 (5.2)	348 (4.6)	409 (5.1)	0.1042				
Meningioma	1 600 (5.3)	336 (4.7)	397 (5.1)	0.1117				
All malignant	10 557 (70.4)	2,697 (69.6)	2,604 (68.5)	0.0665	0.99 [0.97-1.00]	0.381	0.92 [0.86-0.99]	0.047
Glioblastoma	7 849 (76.7)	2 092 (75.5)	1 959 (74.2)	0.0256	0.93 [0.85-1.03]	0.171	0.92 [0.83-1.01]	0.085
Diffuse astrocytoma/anaplastic astrocytoma	1 291 (72.5)	282 (72.5)	312 (74.1)	0.7917				
Oligodendroglioma	569 (64.6%)	113 (66.9%)	113 (62.8%)	0.7206				
**Chemotherapy**								
All tumors	9 611 (20.5)	2 494 (25.6)	2 355 (19.8)	<0.001	1.02 [0.94-1.09]	0.663	0.92 [0.86-0.99]	0.036
All benign	10 (0.03)	4 (0.1)	3 (0.04)	0.5823				
Meningioma	9 (0.03)	4 (0.1)	1 (0.01)	0.3576				
All malignant	9 601 (64.0)	2 490 (64.3)	2 352 (61.9)	0.0359	1.00 [0.99-1.02]	0.701	0.98 [0.86-0.99]	0.033
Glioblastoma	7 326 (71.6)	1 998 (72.1)	1 850 (70.1)	0.2275				
Diffuse astrocytoma/anaplastic astrocytoma	1 223 (68.7)	267 (68.6)	291 (69.1)	0.9829				
Oligodendroglioma	572 (64.9%)	111 (65.7%)	112 (60.0%)	0.3475				

The number of patients who received radiation therapy for glioblastomas decreased in 2020 and 2021, compared to 2016-2019, on univariate analysis (**Table 2**). All patients with malignant tumors saw a minimal increase in chemotherapy treatment in 2020, compared to pre-COVID, which then decreased following 2021 (2016-2019: 64.0%, 2020: 64.3%, 2021: 61.9%, *P* = 0.0359) (**Table 2**). On multivariate analysis, all malignant tumors (OR[95%CI]: 0.98 [0.86-0.99]) had a decrease in chemotherapy treatment in 2021, compared to pre-COVID years (**Table 2**).

Changes in treatment stratified by age categories, sex, and race are displayed in **Supplementary Table 2**. While there was no change in treatment patterns for female patients, males experienced an increase in radiation therapy (2016-2019: 38.5%, 2020: 39.8%, 2021: 36.8%, *P* = 0.014) and chemotherapy (2016-2019: 33.1%, 2020: 34.5%, 2021: 31.1%, *P* = 0.003) during 2020, which then dropped in 2021. White patients (2016-2019: 22.2%, 2020: 23.4%, 2021: 21.6%, *P* = 0.012), Asian/Pacific Islanders (2016-2019: 15.2%, 2020: 20.4%, 2021: 14.9%, *P* < 0.001), and patients above the age of 65 years (2016-2019: 15.0%, 2020: 16.8%, 2021: 14.6%, *P* < 0.001) experienced a similar pattern in chemotherapy. Asian/Pacific Islander patients (2016-2019: 15.2%, 2020: 20.4%, 2021: 14.9%, *P* = 0.001) along with the elderly (2016-2019: 29.0%, 2020: 30.5%, 2021: 30.0%, *P* = 0.047) experienced increased surgery during the peak-COVID era of 2020. Asian/Pacific Islander patients also experienced increased radiation therapy in 2020 (2016-2019: 21.8%, 2020: 25.7%, 2021: 20.9%, *P* = 0.021).

### Trends in Time from Diagnosis to Treatment

There were no significant changes in the time from diagnosis to treatment for benign brain tumors. On univariate analysis, there was a significant decrease in the number of days from all malignant brain tumor diagnosis to first treatment in 2020 and 2021, compared to pre-COVID (2016-2019: 3[0-13] days, 2020: 2[0-11] days, 2021: 2[0-12] days, *P* < 0.001) (**Table 3**). This trend also existed for glioblastoma patients (2016-2019: 3[0-9] days, 2020: 2[0-7] days, 2021: 2[0-8] days, *P* < 0.001).

**Table 3.** Number of days from primary brain tumor diagnosis to initial treatment for pre-COVID years, 2020, and 2021. Linear regression analysis of days from primary brain tumor diagnosis to first treatment for GBM from pre-COVID to 2020 and 2021 is shown for treatments with a *P*-value < 0.10 in the univariate analysis. Covariates include age at diagnosis, sex, and race. Reference year was 2016-2019.

	2016-2019 (*n* = 14 996)	2020 (*n* = 3 875)	2021 (*n* = 3 801)		2020 (*n* = 3 875)		2021 (*n* = 3 801)	
Tumor type	Median [IQR] days			*P*-value	Estimate [95% CI]	*P*-value	Estimate [95% CI]	*P*-value
All tumors	29.40 (68.5)	28.36 (58.3)	27.57 (63.5)	0.141				
All benign	54.42 (96.1)	54.10 (77.7)	52.42 (88.1)	0.39				
Meningioma	54.93 (96.2)	55.11 (89.1)	53.03 (78.28)	0.463				
All malignant	3 [0-13]	2 [0-11]	2 [0-12]	<0.001	−0.51 [−1.2 to −0.1]	0.402	−0.05 [−0.67 to −0.57]	0.932
Glioblastoma	3 [0-9]	2 [0-7]	2 [0-8]	<0.001	−1.25 [−1.71 to −0.78]	0.006	0.86 [0.4-1.32]	0.063
Diffuse astrocytoma/anaplastic astrocytoma	6 [0-30]	4 [0-31.75]	5 [0-28]	0.183				
Oligodendroglioma	4.5 [0-29.5]	5 [0-30]	5 [0-30.5]	.769				

Multivariate linear regression similarly revealed that there were a shorter number of days from glioblastoma diagnosis to first treatment in 2020 compared to pre-COVID (estimate [95%CI]: −1.25 [−1.71 to −0.78], *P* = 0.006) (**Table 3**).

## Discussion

During the COVID-19 pandemic, there were significant alterations in the diagnoses and treatments for all neoplasms due to drastic changes in healthcare system priorities, quarantine restrictions, and patient behavior, among other systemic factors.^9,10^ Overwhelmed with the influx of COVID-19 patients, many hospitals postponed or cancelled elective procedures, including routine screenings and nonurgent surgeries.^23,24^ Moreover, fewer patients sought medical care for early and nonspecific symptoms, such as headaches, nausea, and neurological changes.

These disruptions are echoed through a significantly altered ability to diagnose and treat all primary brain tumor patients. Although our results showed a decrease in the age-adjusted incidence of all primary brain tumors between 2016 and 2021, we found no significant change in the relative incidence of primary malignant brain tumor diagnosed from before and after the peak COVID-19 pandemic. This trend was most likely propelled by a decrease in the number of primary benign brain tumors diagnosed during this time period, though statistically insignificant. The malignant brain tumor patient population underwent a quicker time to treatment from diagnosis and were more likely to receive chemotherapy over radiation, the latter likely to mitigate COVID-19 exposure. Overall, this constellation of findings undercovers new trends with regards to behavior and healthcare delivery in the wake of COVID-19 pandemic where the incidence and treatment of primary brain tumor, especially malignant tumors, were not severely affected as these tumors were more likely to be prioritized during COVID-19.

### Incidence of Primary Brain Tumors

During the height of the pandemic in 2020, there was a drop in overall primary brain tumor diagnoses, composed of both malignant and benign primary brain tumors, presumed to result from hesitancy by patients to seek care as well as the reduction of routine scans during this uncertain time.^24,25^ This decrease in incidence for overall brain tumors may be driven by the decrease in incidence of primary benign brain tumors, which comprise the large majority of primary brain tumors, especially in our data, and are usually slower growing and more asymptomatic, compared to their malignant counterparts.^26^ This change is not restricted to brain tumor patients nor is unique to our findings. In a study conducted by Cairns et al., there was a 44% reduction in overall screening mammograms and 21% reduction in diagnostic mammograms from 2019 to 2020.^27^ In fact, there were 34 000 fewer cases of breast cancer than expected in 2020.^28^ This same finding is also seen across other cancer types such as melanoma^29^ and prostate cancer.^30^ This may be due to national news channels emphasizing hospital systems being overwhelmed by the influx of COVID-19 affected patients. Many citizens were urged to refrain from seeking hospital care barring an emergency.^31,32^ Given that typically brain tumors fail to manifest with symptoms that would mimic COVID-19 or an emergency, and rather present as mild headaches and nonspecific neurological symptoms, many patients likely delayed seeking care and adhered to news outlets’ and government officials’ advice to avoid the resource-limited and contagious healthcare system.

Although the incidence of all brain tumors dropped overall, malignant primary brain tumor incidence remained stable to prepandemic levels, mirroring findings to other severe cancer types like pancreatic cancer or metastatic breast cancer which showed negligible differences in incidence during the pandemic.^33,34^ This stability may be explained by appropriate triaging by medical staff and hospital systems during the pandemic, where emergent diseases, such as malignant brain tumors, were prioritized for brain surgery, despite many operating rooms being utilized for COVID-19 care. Additionally, malignant brain tumors, which generally present with concerning neurological symptoms more rapidly, urged patients to outweigh the risk of COVID-19 exposure to seek prompt care. At the same time, with a decrease in routine screening and subtle symptomology of benign neurological neoplasms, it remains plausible that patients with benign brain tumors experienced a delay of care because of the healthcare system changes and their own fears in contracting COVID-19. From the clinician’s perspective, patients presenting with a malignant neoplasm would become a priority to see sooner through telemedicine or in person for a more comprehensive visit.

In their analysis of the early months of 2020, Cioffi et al. had found that black patients had a larger decrease in incidence of brain tumor diagnosis than white patients.^19^ This can be explained by Islam et al.’s finding that the first wave of COVID, defined from April to June 2020, had the highest rates of cancer treatment delay or discontinuation and subsequently decreased for both black and white identifying patients.^35^ In our study, we found no differences in age-adjusted incidence rate among different racial groups, except for American Indians/Alaskan Natives.

### Time to Treatment

The COVID-19 pandemic forced healthcare professionals to amend their contemporary treatment strategies, as the uncertainty of the pandemic required them to prioritize certain patients to receive treatments in a resource-limited setting.^36^ Studies had shown that patient level factors, in addition to a cancer diagnosis, led to increased odds of treatment delay during the pandemic.^37^ Surgical delays for cancer patients, such as those with renal masses, had been associated with a worsened overall survival.^38^ Interestingly, despite significant strains on the healthcare system, our data demonstrated minimal treatment delays for all brain tumor types. In fact, the most aggressive brain tumors, like Glioblastoma (GBM), were treated more quickly during the pandemic, likely due to the prioritization of malignant cases and reduction of non-urgent surgeries as recommended by the American Association of Neurological Surgeons (AANS) during that time.^39^ This lack of change in time to treatment for brain tumor patients is echoed in the findings by Normal et al. who found that COVID-19 did not lead to worsened outcomes for neuro-oncology patients.^40^

Additionally, Llanos et al. had found that alongside disproportionate burdens of COVID-19 diagnoses among racial and ethnic minority patients, these groups also suffered from a higher rate of cancer treatment delays or discontinue during the pandemic.^41^ In our findings, we found no such difference in incidence of primary brain tumor and time from diagnosis to proper treatment amongst different racial and ethnic groups. However, we did find that Asian/Pacific Islander patients had increased rates of surgery, radiation, and chemotherapy in 2020 compared to 2016-2019.

### Treatment Patterns

Treatment patterns for surgery, chemotherapy, and radiation shifted noticeably from prepandemic to peak-COVID periods. While healthcare systems saw a substantial decrease in overall surgical volume due to a prioritization of emergent cases and postponement of elective cases based on CDC recommendations,^42^ there was a slight increase in surgical interventions for malignant tumors, particularly GBM. This shift likely resulted from surgical suites predominantly focusing on emergent cases to minimize hospital traffic and reduce strain on the healthcare system. From a neurosurgical perspective, a similar approach was adopted to treat highly symptomatic patients and those with aggressive tumors. This prioritization is reflected in the consistent, even slight increase in proportion of GBM cases during the pandemic along with the significant decrease in the time from diagnosis to surgical intervention for GBM patients. Further, this is backed by the AANS guidelines during the pandemic that created a tier-based system on prioritization of neurosurgical operations. Although the guidelines do not specify which pathologies fall into each tier, it can be inferred that GBM, due to its severity, likely fell into the third tier, which means physicians were advised to swiftly proceed without delay.^43^ By 2021, as elective surgeries resumed and ORs became busier, surgical rates began returning to prepandemic levels.^44^ For patients with less severe tumor types, such as meningiomas, there were no significant changes in time to surgery, highlighting the continued efforts to maintain standard care for all brain tumor patients but making an intentional effort to prioritize malignant tumors like GBM.

Concurrently, there was a significant reduction in radiation therapy for those with aggressive tumors as hospitals sought for alternative solutions to minimize patient exposure to COVID-19 by reducing frequent hospital visits required for radiation therapy. Practices reported treating only 68% of their typical radiation therapy volume, largely due to postponing treatments for lower-risk patients, based on a survey of radiation oncologists throughout the US, released by the American Society for Radiation Oncology.^45^ However, it is noted in this national survey that radiation oncology practices did not severely increase treatment postponement for CNS high-grade gliomas and glioblastomas.

Additionally, radiation centers reported resorting to increased use of hypofractionated regimens, delivering higher doses in a shorter period of time, to decrease treatment time and COVID-19 exposure.^46,47^ A new hypofractionation regimen was derived at an institution specifically for glioblastoma patients so that patients could continue receiving radiation treatment while minimizing risk of COVID-19 infection.^48^ However, as numbers returned to prepandemic levels in 2021, this 2020 reduction was likely intentional in reducing hospital traffic and patient exposure to COVID-19.

### Limitations

We recognize several limitations in our study. First, the SEER database may have variation in its reporting and data entry across different centers. Additionally, when calculating time from diagnosis to treatment, the treatment type was not specified to understand if curative or for symptom management. Data included in the dataset was incomplete and required removing these patients from our analysis. Next, SEER does not include patient comorbidities, and other patient specific variables, which may have influenced choice to offer patients treatment during the pandemic. Our study excludes patients who did not have access to medical care and thus were not captured in the SEER registry. Also, 2022 SEER data was not released at time of analysis. Therefore, we are unable to assess changes in trends following 2021. Likewise, we cannot assess trends between months as SEER data is limited to year of diagnosis. Lastly, SEER only captures data from 26.5% of the United States population, and therefore our findings might not be representative of the country and include geographic biases.^49^

## Conclusion

The COVID-19 pandemic disrupted the diagnosis and treatment of all primary brain tumors, likely driven delays in the benign etiology. Despite that, malignant tumors, like glioblastoma, maintained a stable incidence due to their aggressive symptoms. Treatment patterns shifted, with quicker intervention for malignant cases and a temporary reliance on chemotherapy over radiation to potentially reduce patient exposure to the virus or to compensate for changes in personnel and resource allocation. These findings indicate that, overall, the management of malignant brain tumors during the COVID-19 pandemic remained effective, with a continued focus on delivering high-quality care and prioritizing patients with primary malignant brain tumors. Resilience and adaptability in clinical practices were demonstrated in neurosurgical oncology practices, despite the challenges posed by the pandemic.

## Supplementary Material

vdaf181_suppl_Supplementary_Table_1

vdaf181_suppl_Supplementary_Table_2

## Data Availability

No new data were generated or analyzed in support of this research.
