# Supplementary material for: Impact of the COVID-19 pandemic on primary brain tumor incidence and management: Decisions that went right
Source: Neurooncol Adv. 2025 Aug 16;7(1):vdaf181. doi: 10.1093/noajnl/vdaf181 (PMC12448709; doi:10.1093/noajnl/vdaf181)
Supplement: vdaf181_suppl_Supplementary_Table_1 [file vdaf181_suppl_supplementary_table_1.docx]

**Supplementary Table 1:** SEER Brain Tumor Pathology Labels and Descriptions

| **Category** | **Primary Site** | **Behavior** | **Histology** | **Recode value** |
| --- | --- | --- | --- | --- |
| **Malignant Brain/ONS** | 700-729 | 3 | Excluding 9050–9055, 9140, 9590–9992 | 1-15 |
| **1. Glioma** | 700-729 | 3 | 9380–9385, 9391–9460 | 1-10 |
| 1.1 Diffuse astrocytoma and anaplastic astrocytoma | 700-729 | 3 | 9400-9401,9410-9411,9420 | 1 |
| 1.2 Glioblastoma | 700-729 | 3 | 9440-9442,9445 | 2 |
| 1.3 Diffuse midline glioma, H3 K27M-mutant | 700-729 | 3 | 9385 | 3 |
| 1.4 Oligodendroglioma | 700-729 | 3 | 9450-9451 | 4 |
| 1.5 Oligoastrocytoma | 700-729 | 3 | 9382 | 5 |
| 1.6 Other astrocytic tumors | 700-729 | 3 | 9421,9424-9425 | 6 |
| 1.7 Astroblastoma | 700-729 | 3 | 9430 | 7 |
| 1.8 Ependymal tumors | 700-729 | 3 | 9391-9393,9396 | 8 |
| 1.9 Glioma, unspecified | 700-729 | 3 | 9380 | 9 |
| 1.10 Other | 700-729 | 3 | 9381,9383-9384,9394-9395,9397-9399,9402-9409,9412-9419,9422-9423,9426-9429,9431-9439,9443-9444,9446-9449,9452-9460 | 10 |
| **2. Embryonal tumors** | 700-729 | 3 | 8963,9364,9470-9478,9490,9500-9502,9508 | 11 |
| **3. Meningiomas** | 700-729 | 3 | 9530,9538-9539 | 12 |
| **4. Choroid plexus tumors** | 700-729 | 3 | 9390 | 13 |
| **5. Neuronal and mixed neuronal-glial tumors** | 700-729 | 3 | 9505, 9509 | 14 |
| **6. Other Malignant Brain/ONS** | 700-729 | 3 | Not listed above, excluding 9050–9055, 9140, 9590–9993 | 15 |
| **Non-malignant Brain/ONS** | 700-729 | 0-1 | Excluding 9050–9055, 9140, 9590–9992 | 16-24 |
| **1. Meningiomas** | 700-729 | 0 | 9530-9534,9537 | 16 |
|  |  | 1 | 9538-9539 |  |
| **2. Tumors of the cranial and paraspinal nerves** | 700-729 | 0 | 9540, 9550, 9560, 9571, 9563 | 17 |
|  |  | 1 | 9560 |  |
| **3. Ependymal tumors** | 700-729 | 1 | 9383, 9394 | 18 |
| **4. Choroid plexus tumors** | 700-729 | 0 | 9390 | 19 |
|  |  | 1 | 9390 |  |
| **5. Neuronal and mixed neuronal-glial tumors** | 700-729 | 0 | 9413, 9492-9493, 9509 | 20 |
|  |  | 1 | 8693, 9412, 9505-9506, 9509 |  |
| **6. Mesenchymal, nonmeningothelial tumors** | 700-729 | 0 | 8815,8825,8830,8850,8861,8880,8890,8900,9120,9180,9210,9220 | 21 |
|  |  | 1 | 8815,8821,8825,9161 |  |
| **7. Other astrocytic tumors** | 700-729 | 1 | 9384 | 22 |
| **8. Other gliomas** | 700-729 | 1 | 9431,9444 | 23 |
| **9. Other non-malignant Brain/ONS** | 700-729 | All other combinations, excluding 9050–9055, 9140, 9590–9993 | 24 |  |
| **Malignant tumors of the pineal region** | 753 | 3 | 9362,9395 | 25 |
| **Non-malignant tumors of the pineal region** | 753 | 1 | 9361 | 26 |
| **All Other Cancer Types** | All other combinations | 27 |  |  |
