# Supplementary material for: Impact of the COVID-19 pandemic on primary brain tumor incidence and management: Decisions that went right
Source: Neurooncol Adv. 2025 Aug 16;7(1):vdaf181. doi: 10.1093/noajnl/vdaf181 (PMC12448709; doi:10.1093/noajnl/vdaf181)
Supplement: vdaf181_suppl_Supplementary_Table_2 [file vdaf181_suppl_supplementary_table_2.docx]

**Supplementary Table 2:** Changes in treatment patterns and treatment delay in primary brain tumor patients, stratified by sex, race, and age demographic categories.

| **Treatment** | **2016-2019** | **2020** | **2021** | **P-value** |
| --- | --- | --- | --- | --- |
| **Females** | | | | |
| Surgery | 10,266 (34.6) | 2,471 (34.9) | 2,637 (34.8%) | 0.837 |
| Radiation | 5,613 (18.9) | 1,304 (18.4) | 1,433 (18.9) | 0.644 |
| Chemotherapy | 3,935 (13.3) | 987 (13.9) | 1,022 (13.5) | 0.370 |
| Days from Diagnosis to Treatment  (average ± SD) | 36.7 ± 78.3 | 34.7 ± 72.4 | 35.2 ± 65.3 | 0.346 |
| **Males** | | | | |
| Surgery | 9,089 (53.1) | 2,370 (54.2) | 2,242 (52.2) | 0.173 |
| Radiation | 6,596 (38.5) | 1,741 (39.8) | 1,580 (36.8) | 0.014 |
| Chemotherapy | 5,658 (33.1) | 1,507 (34.5) | 1,333 (31.1) | 0.003 |
| Days from Diagnosis to Treatment  (average ± SD) | 21.1 ± 54.1 | 20.1 ± 51.2 | 20.1 ± 47.2 | 0.583 |
| **White** | | | | |
| Surgery | 15,958 (42.2) | 3,924 (42.8) | 3,978 (41.9) | 0.399 |
| Radiation | 10,432 (27.6) | 2,569 (28.0) | 2,571 (27.1) | 0.334 |
| Chemotherapy | 8,397 (22.2) | 2,141 (23.4) | 2,053 (21.6) | 0.012 |
| Days from Diagnosis to Treatment  (average ± SD) | 28.1 ± 66.3 | 26.1 ± 60.7 | 26.5 ± 55.3 | 0.101 |
| **Black/ African American** | | | | |
| Surgery | 1,575 (35.9) | 400 (35.1) | 393 (35.3) | 0.848 |
| Radiation | 815 (18.6) | 208 (18.2) | 194 (17.4) | 0.672 |
| Chemotherapy | 545 (12.4) | 149 (13.1) | 130 (11.7) | 0.601 |
| Days from Diagnosis to Treatment  (average ± SD) | 38.5 ± 87.3 | 31.3 ± 65.1 | 31.3 ± 60.2 | 0.087 |
| **Asian/Pacific Islander** | | | | |
| Surgery | 1,518 (40.4) | 421 (46.7) | 411 (40.9) | 0.001 |
| Radiation | 820 (21.8) | 232 (25.7) | 210 (20.9) | 0.021 |
| Chemotherapy | 571 (15.2) | 184 (20.4) | 150 (14.9) | <0.001 |
| Days from Diagnosis to Treatment  (average ± SD) | 31.7 ± 65.3 | 36.2 ± 84.5 | 39.2 ± 75.5 | 0.092 |
| **Hispanic/Latino** | | | | |
| Surgery | 3,044 (49.6) | 768 (49.6) | 785 (27.2) | 0.670 |
| Radiation | 1,682 (27.4) | 424 (27.4) | 441 (27.2) | 0.983 |
| Chemotherapy | 1,266 (20.6) | 340 (21.9) | 327 (20.1) | 0.413 |
| Days from Diagnosis to Treatment  (average ± SD) | 34.1 ± 77.6 | 29.2 ± 64.7 | 33.3 ± 65.4 | 0.22 |
| **18-39 Years Old** | | | | |
| Surgery | 2,734 (66.2) | 700 (69.3) | 665 (66.9) | 0.177 |
| Radiation | 1,502 (36.4) | 368 )36.4) | 385 (38.7) | 0.376 |
| Chemotherapy | 1,262 (30.6) | 308 (30.5) | 306 (30.8) | 0.989 |
| Days from Diagnosis to Treatment  (average ± SD) | 28.7 ± 65.1 | 27.1 ± 63.0 | 25.6 ± 51.8 | 0.460 |
| **40-64 Years Old** | | | | |
| Surgery | 9,551 (52.2) | 2,290 (52.2) | 2,263 (51.8) | 0.812 |
| Radiation | 5,894 (32.2) | 1,410 (32.3) | 1,375 (31.5) | 0.624 |
| Chemotherapy | 4,698 (25.7) | 1,168 (26.8) | 1,100 (25.2) | 0.200 |
| Days from Diagnosis to Treatment  (average ± SD) | 31.2 ± 70.4 | 29.9 ± 66.1 | 31.8 ± 62.3 | 0.600 |
| **65+ Years Old** | | | | |
| Surgery | 7,070 (29.0) | 1,851 (30.5) | 1,951 (30.0) | 0.047 |
| Radiation | 4,813 (19.7) | 1,267 (20.9) | 1,253 (19.2) | 0.063 |
| Chemotherapy | 3,651 (15.0) | 1,018 (16.8) | 949 (14.6) | <0.001 |
| Days from Diagnosis to Treatment  (average ± SD) | 27.3 ± 67.2 | 25.0 ± 60.2 | 25.4 ± 55.2 | 0.191 |
